# 

**Published:** 1813-07

**Authors:** 


### THE MEDICAL AND PHYSICAL JOURNAL.

CONDUCTED BY

SAMUEL FOTHERGILL, M.D.

OF THE ROYAL COLLEGE OF PHYSICIANS; PHYSICIAN TO THE ASYLUM FOR FEMALE ORPHANS; AND TO THE WESTMINSTER GENERAL, AND THE WESTERN, DISPENSARIES.

VOL. XXX.

FROM JULY TO DECEMBER, 1813.

Et quoniam variant morbi, variabimus artes;

Mille mali species, mille salutis erunt.

*LONDON:*

PRINTED FOR RICHARD PHILLIPS; BY J. ADLARD, 23, BARTHOLOMEW-CLOSE, AND 39, DUKE-STREET, SMITHFIELD; AND SOLD BY J. SOUTER, NO. 1, PATERNOSTER-ROW.

1814.

5136

W
1
ME. 182

[Entered at Stationers' Hall.]

### MONTHLY CATALOGUE OF MEDICAL BOOKS.

A PRACTICAL Synopsis of Cutaneous Diseases, according to the Arrangement of Dr. Willan; exhibiting a concise View of the Diagnostic Symptoms, and the Method of Treatment. By Thomas Bateman, M.D. F.L.S. 8vo.—Longman and Co.

The Influence of Tropical Climates, more especially the Climate of India, on European Constitutions. By James Johnson. 8vo.—Stockdale.

The Philosophy of Medicine, being Medical Extracts on the Nature and Preservation of Health, and on the Nature and Removal of Disease. By Robert John Thornton, M.D. In 2 vols.—Sherwood.

Practical Remarks on the Diseases resembling Syphilis, with Cases. By John Whitsed. 8vo.—Crosby and Co.

An Essay on the Utility of Blood-letting in Fever; illustrated by numerous Cases: with some Inquiry into the Seat and Nature of this Disorder. By Thomas Mills, M.D. 8vo.—Longman and Co.

Experimental Researches concerning the Philosophy of Permanent Colours, and the best Means of producing them, by Dyeing, Calico Printing, &c. By Edward Bancroft, M.D. 2 vols. 8vo.—Cadell and Co.

Practical Observations on the Use and Abuse of Cold and Warm Sea Bathing in various Diseases, particularly in Scrofulous and Gouty Cases. By John Gibney, M.D. 8vo.—Underwood.

An Inquiry into the Laws of Animal Life, being an Analysis of the Principles of Medical Science. By J. M. Park, M.B. 8vo.—Underwood.

### *Scientific Books in hand, or in the Press.*

Mr. Stevenson has in the press a second edition of his Treatise on Morbid Sensibility of the Eye.

Mr. Leslie, Professor of Mathematics in the University of Edinburgh, is just upon the point of publishing a View of Experiments and Instruments depending on the relation of Air to Heat and Moisture.

Capt. Laskey has in the press A Scientific Description of the Rarities in that magnificent collection the Hunterian Museum, now deposited at the College of Glasgow. It is intended to comprise the rare, curious, and valuable articles, in every department of Art, Science, and Literature, contained in that great repository. This work may be expected to appear early in July.

Mr. Henry Alexander, surgeon, will shortly publish a Comparative View of the different Modes of operating for the Cataract.

---

### *NOTICES TO CORRESPONDENTS.*

*A Drawing of an ingenious Footstool, by Mr. Jennens, is received, and will appear in our next Number.*

*Several communications on Medical Reform have reached us, but the subject has already occupied so much space in the Journal, that we hope our friends will excuse our not inserting any more papers on this head for the present.*

*Communications from Dr. Prout; Dr. Kinglake; &c. &c. in our next.*

